# Electrophysiological Abnormalities in VLCAD Deficient hiPSC-Cardiomyocytes Can Be Improved by Lowering Accumulation of Fatty Acid Oxidation Intermediates

**DOI:** 10.3390/ijms21072589

**Published:** 2020-04-08

**Authors:** Suzan J. G. Knottnerus, Isabella Mengarelli, Rob C. I. Wüst, Antonius Baartscheer, Jeannette C. Bleeker, Ruben Coronel, Sacha Ferdinandusse, Kaomei Guan, Lodewijk IJlst, Wener Li, Xiaojing Luo, Vincent M. Portero, Ying Ulbricht, Gepke Visser, Ronald J. A. Wanders, Frits A. Wijburg, Arie O. Verkerk, Riekelt H. Houtkooper, Connie R. Bezzina

**Affiliations:** 1 Laboratory Genetic Metabolic Diseases, Amsterdam UMC, University of Amsterdam, Amsterdam Gastroenterology and Metabolism, Amsterdam Cardiovascular Sciences, 1105 AZ Amsterdam, The Netherlands; s.j.knottnerus@amsterdamumc.nl (S.J.G.K.); r.wust@vu.nl (R.C.I.W.); jeannette.bleeker@gmail.com (J.C.B.); s.ferdinandusse@amsterdamumc.nl (S.F.); l.ijlst@amsterdamumc.nl (L.I.); G.Visser-4@umcutrecht.nl (G.V.); r.j.wanders@amc.uva.nl (R.J.A.W.); 2 Department of Paediatric Metabolic Diseases, Wilhelmina Children’s Hospital, University Medical Center Utrecht, 3584 EA Utrecht, The Netherlands; 3 Department of Clinical and Experimental Cardiology, Heart Center, Amsterdam Cardiovascular Sciences, Amsterdam UMC, University of Amsterdam, 1105 AZ Amsterdam, The Netherlands; i.mengarelli@amsterdamumc.nl (I.M.); a.baartscheer@amsterdamumc.nl (A.B.); r.coronel@amsterdamumc.nl (R.C.); v.m.portero@amsterdamumc.nl (V.M.P.); a.o.verkerk@amsterdamumc.nl (A.O.V.); 4 Institute of Pharmacology and Toxicology, Technische Universität Dresden, 01069 Dresden, Germany; kaomei.guan@tu-dresden.de (K.G.); wener.li@tu-dresden.de (W.L.); xiaojing.luo@tu-dresden.de (X.L.); yingulbricht@gmail.com (Y.U.); 5 Department of Paediatric Metabolic Diseases, Emma Children’s Hospital, Amsterdam UMC, University of Amsterdam, 1105 AZ Amsterdam, The Netherlands; f.a.wijburg@amsterdamumc.nl; 6 Department of Medical Biology, Amsterdam Cardiovascular Sciences, Amsterdam UMC, University of Amsterdam, 1105 AZ Amsterdam, The Netherlands

**Keywords:** VLCADD, arrhythmias, hiPSC, acylcarnitines

## Abstract

Patients with very long-chain acyl-CoA dehydrogenase deficiency (VLCADD) can present with life-threatening cardiac arrhythmias. The pathophysiological mechanism is unknown. We reprogrammed fibroblasts from one mildly and one severely affected VLCADD patient, into human induced pluripotent stem cells (hiPSCs) and differentiated these into cardiomyocytes (VLCADD-CMs). VLCADD-CMs displayed shorter action potentials (APs), more delayed afterdepolarizations (DADs) and higher systolic and diastolic intracellular Ca^2+^ concentration ([Ca^2+^]_i_) than control CMs. The mitochondrial booster resveratrol mitigated the biochemical, electrophysiological and [Ca^2+^]_i_ changes in the mild but not in the severe VLCADD-CMs. Accumulation of potentially toxic intermediates of fatty acid oxidation was blocked by substrate reduction with etomoxir. Incubation with etomoxir led to marked prolongation of AP duration and reduced DADs and [Ca^2+^]_i_ in both VLCADD-CMs. These results provide compelling evidence that reduced accumulation of fatty acid oxidation intermediates, either by enhanced fatty acid oxidation flux through increased mitochondria biogenesis (resveratrol) or by inhibition of fatty acid transport into the mitochondria (etomoxir), rescues pro-arrhythmia defects in VLCADD-CMs and open doors for new treatments.

## 1. Introduction

Very long-chain acyl-CoA dehydrogenase deficiency (VLCADD) (OMIM 201475), an autosomal recessive inborn error of metabolism, caused by pathogenic mutations in the *ACADVL* gene, is a mitochondrial long-chain fatty acid oxidation (lcFAO) disorder [1,2]. Patients with VLCADD may develop hypoglycemia, rhabdomyolysis, cardiomyopathy and cardiac arrhythmias [3,4,5,6]. Because some of these clinical signs and symptoms can be fatal early in life and are at least partly preventable, VLCADD has been included in newborn screening programs worldwide [7]. There is a marked variety in the development of clinical symptoms among patients [8]. Cardiomyopathy and rhythm disturbances occur almost exclusively in patients with a low residual lcFAO flux (<10%) and are not prevented by early dietary treatment (avoidance of fasting) [7]. Mechanisms underlying cardiac arrhythmias in VLCADD, which can occur even in the absence of structural cardiac malformation [4,9] are not well understood. Two putative pathophysiological mechanisms have been proposed for the cardiac phenotype—(1) energy shortage due to deficient adenosine triphosphate (ATP) synthesis from lcFAO, and (2) accumulation of lcFAO intermediates including long-chain acylcarnitines (LCACs) [9,10]. Of note, in acute myocardial ischemia, rapid LCAC accumulation also occurs [11] and for this reason the modulating effects of LCACs on cardiac action potentials (APs) and ion currents have been widely studied [12,13,14,15,16,17]. Exogenously added LCACs can induce a variety of electrophysiological alterations in ventricular myocytes, including AP shortening [13], cellular uncoupling [18] and delayed afterdepolarizations (DADs) [13,14], the latter representing an important cellular mechanism for arrhythmias [19,20].

Previous work showed that resveratrol increases lcFAO flux and decreases LCAC accumulation in fibroblasts of VLCADD patients with milder phenotypes [21,22]. In these studies, the effects of resveratrol were thought to be mediated by an increase in the amount of mutated VLCAD protein via sirtuin 1 activation [21]. In cells with null-type mutations (e.g., nonsense, frameshift, truncating mutations) in *ACADVL*, resveratrol cannot increase lcFAO flux, considering the virtually absent VLCAD activity [21,23]. Decrease in potentially toxic LCAC accumulation can also be acquired by incubating cells with etomoxir, a pharmacological inhibitor of carnitine palmitoyltransferase 1 (CPT1) [24], one of the enzymes that is involved in the mitochondrial import of long-chain fatty acids by converting long-chain acyl-CoAs to LCACs [25].

Development of human induced pluripotent stem cell (hiPSC) technology provides the opportunity to generate VLCADD patient-specific cardiomyocytes (VLCADD-CMs) and to study diseases on a cell-type specific level [26]. In order to progress towards prevention or therapeutic interventions for VLCADD-induced cardiac arrhythmias, we generated hiPSC-CMs from two patients harboring distinct *ACADVL* genetic defects and studied the underlying mechanism of the pro-arrhythmic phenotype using compounds affecting different aspects of lcFAO biochemistry, namely resveratrol and etomoxir.

## 2. Results

### 2.1. VLCADD Patients

The biochemical and clinical characteristics of the patients are described in Table 1. Patient 1 had a residual lcFAO flux in fibroblasts that was 32% of control values suggesting a mild phenotype. She presented for the first time at the age of 15 months with severe hypoglycemia (0.2 mmol/L) and hypothermia (35.9 °C), during an infection. ECGs were not obtained during this episode. At age 15 years, a routine Holter-ECG showed no abnormalities. With dietary adjustments, that is, avoidance of fasting, no severe episodes occurred after the initial presentation. Patient 2 had a lcFAO flux of 7%, indicative of a severe VLCADD phenotype. She presented at the age of 6 weeks with hypoglycemia (1.7 mmol/L), convulsions and tachycardia. At age of 2 months, echocardiography revealed a hypertrophic cardiomyopathy. During childhood, she had frequent metabolic derangements and episodes of relapsing cardiomyopathy. No ECGs during metabolic derangement were available. In a stable condition at age 15 years, the patient showed aspecific repolarization abnormalities on the ECG (flat ST-T segments in the inferior and left lateral leads) and early repolarizations in the inferior leads. A routine Holter-ECG at age 18 years showed single atrial and ventricular ectopy and a Mobitz II AV-block during sleep.

### 2.2. Generation of Patient-Specific VLCADD-CM

Fibroblasts from both patients and from a healthy control were reprogrammed to generate hiPSC lines (iVLCADD1 from Patient 1, iVLCADD2 from Patient 2 and iCTRL (Figure S1)). These lines were differentiated to cardiomyocytes (iVLCADD1-CMs, iVLCADD2-CMs and iCTRL-CMs; details in Material and Methods) and subjected to biochemical analyses as well as single cell electrophysiological and [Ca^2+^]_i_ analysis.

LcFAO flux is expressed as percentage of the mean flux in healthy control cell lines measured in the same experiment. VLCAD activities are expressed as percentage of reference mean. Detection limit for VLCAD activity in lymphocytes and fibroblasts was 0.15 nmol/min/mg and 0.06 nmol/min/mg, respectively. ECG: electrocardiogram. lcFAO flux: long-chain fatty acid oxidation flux. Maximal creatine kinase is the highest value reported in the medical history of the patient.

### 2.3. VLCADD-CMs Accumulate Long-Chain Acylcarnitine Species

**Figure 1:**
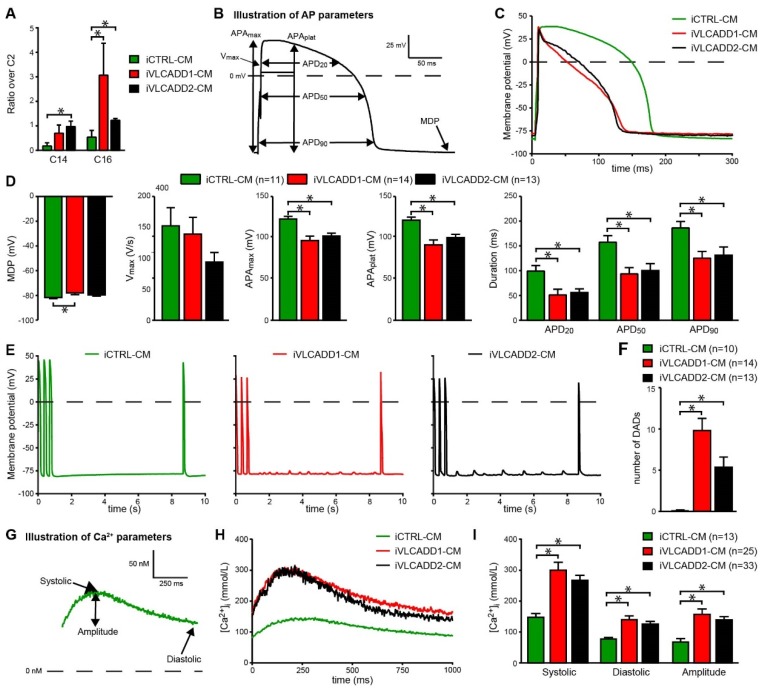
Electrophysiological abnormalities in very long-chain acyl-CoA dehydrogenase deficiency (VLCADD) human induced pluripotent stem cell- cardiomyocytes (hiPSC-CMs). (**A**) Increased C14/C2 carnitine and C16/C2 carnitine ratio in medium of iVLCADD1-CMs and iVLCADD2-CMs compared to control cardiomyocytes (iCTRL-CM) after 96 h culturing with palmitate loading. Data expressed as mean + SD (*n* = 3). Statistical analysis was performed using a two-way ANOVA with Sidaks multiple comparisons test. * = *p* < 0.05. (**B**) Analyzed action potential (AP) parameters. (**C**) Representative APs paced at 1 Hz. (**D**) Average AP parameters. (**E**) Typical delayed afterdepolarizations (DADs) measured during an 8 s pause after fast pacing (3 Hz). After this pause, a single AP was evoked to confirm that no early afterdepolarizations occurred. (**F**) Average number of DADs. Electrophysiology data are expressed as mean+SEM of individual measured cells (*n* = 11–13). (**G**) Analyzed parameters in Ca^2+^ transients. (**H**) Representative Ca^2+^ transients of Indo-1 loaded hiPSC-CMs paced at 1 Hz. (**I**) Average [Ca^2+^]i (mean+SEM) * = *p* < 0.05. Statistical analysis was assessed with one-way ANOVA with Tukey’s multiple comparisons test.

To confirm the lcFAO deficiency in the VLCADD hiPSC-CMs, we measured LCAC accumulation in the medium after supplementation with stable isotope-labeled [U−^13^C]-palmitic acid, as a diagnostic marker for VLCADD. The ratio of LCACs over C2-carnitine was markedly increased in the incubation medium of both iVLCADD1-CMs and iVLCADD2-CMs (Figure 1A), confirming that these cells retained their VLCAD deficiency. We also tested if cells accumulated LCAC under regular hiPSC-CM culture conditions. These cells were routinely cultured in medium containing a relatively low amount of carnitine and fatty acids (≈11 µmol/L and ≈30 µmol/L respectively) but even under these conditions LCAC levels were increased in VLCADD-CMs compared to iCTRL-CMs (Figure S2).

### 2.4. VLCADD-CMs Exhibit Shortened Action Potentials and Delayed after Depolarizations

Next, we assessed the implications of VLCADD on various AP parameters (Figure 1B) and susceptibility to early afterdepolarization (EAD) and DAD generation in dissociated single hiPSC-CMs. Representative APs are shown in Figure 1C and average AP parameters are shown in Figure 1D. Maximal AP amplitude (APAmax), AP plateau amplitude (APAplat) and AP duration measured at 20%, 50% and 90% of repolarization (APD_20_, APD_50_ and APD_90_, respectively) were significantly lower in the two VLCADD-CM lines compared to iCTRL-CMs (Figure 1D). The susceptibility to occurrence of DADs was tested by applying a fast pacing stimulus followed by an 8-s pause. After the 8-s pause, a single AP was evoked to test the occurrence of EADs. EADs were never observed, however, we observed many DADs in VLCADD-CMs. Figure 1E,F show typical traces and the average number of DADs, respectively. In both VLCADD-CMs the number of DADs was markedly increased compared to control (Figure 1F).

### 2.5. VLCADD-CMs Show Increased Ca^2+^ Concentration

Using fluorescence measurements, we characterized various Ca^2+^ transient parameters (Figure 1G) and found a higher systolic and diastolic intracellular Ca^2+^ concentration ([Ca^2+^]_i_) as well as an increased transient amplitude in iVLCADD1-CMs and iVLCADD2-CMs compared to iCTRL-CMs (Figure 1H–I). Collectively, these results demonstrate that hiPSC-CMs from VLCADD patients exhibit increased LCAC accumulation and that [Ca^2+^]_i_ is increased, accompanied by AP shortening and presence of DADs.

### 2.6. Resveratrol Improves Biochemical and Electrophysiological Derangements in VLCADD-CM of Patient 1

**Figure 2:**
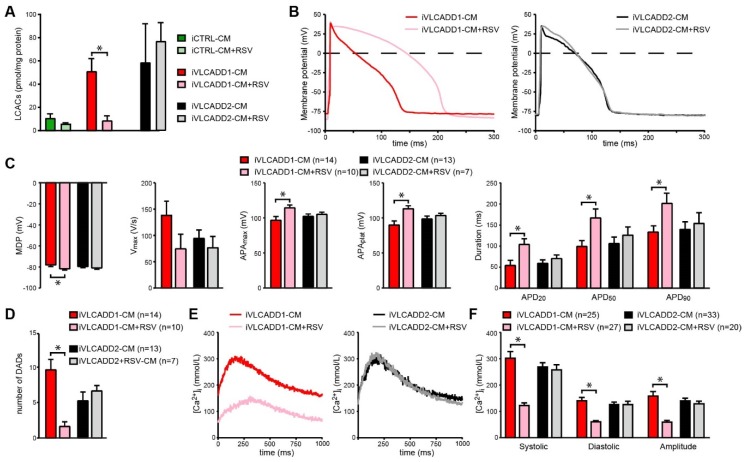
Rescue of electrophysiological abnormalities in iVLCADD1-CMs after pre-incubation with Resveratrol (RSV; 50 µmol/L). (**A**) Intracellular acylcarnitine levels in iVLCADD1-CMs, iVLCADD2-CMs and iCTRL-CMs after RSV incubation for 96 h. Sum of long-chain acylcarnitines (LCACs) (C12-, C14-, C14:1-, C16-, C16:1-, C18-, C18:1-carnitine). Values represent mean + SD in two biological replicates. Statistical analysis was performed using a two-way ANOVA with Tukey’s multiple comparisons test. * = *p* < 0.05. (**B**) Representative APs paced at 1 Hz. (**C**) Average AP parameters after RSV or vehicle (dimethyl sulfoxide (DMSO)) pre-incubation. (**D**) Number of DADs in absence and presence of RSV. (**E**) Representative Ca^2+^ transients of Indo-1 loaded hiPSC-CMs paced at 1 Hz in presence of RSV. (**F**) Average [Ca^2+^]_i_ after RSV or vehicle (DMSO) pre-incubation. For AP analyses and [Ca^2+^]_i_ data is indicated as mean+SEM. * = *p* < 0.05. Number of cells is indicated in the figure. Statistical analysis was assessed with one-way ANOVA with Tukey’s multiple comparisons test.

In line with expectations based on genotype (Patient 1 is compound heterozygous for missense mutations which generate a mitochondrial enzyme with residual activity, whereas Patient 2 is homozygous for a truncating mutation which eliminates the enzyme activity), incubation with the mitochondrial booster resveratrol induced a strong increase in lcFAO flux in cultured fibroblasts from patient 1 but not in patient 2 (Figure S3). Therefore, we hypothesized that resveratrol would reduce LCAC accumulation and would mitigate the electrophysiological alterations only in iVLCADD1-CMs. Indeed, we found that LCAC accumulation was reduced in iVLCADD1-CMs but not in iVLCADD2-CMs (Figure 2A) after 48h pre-incubation with resveratrol. Strikingly, resveratrol also increased the AP amplitude and prolonged the AP in iVLCADD1-CMs (Figure 2B,C). In addition, [Ca^2+^]_i_ and the number of DADs was significantly reduced after resveratrol treatment in iVLCADD1-CMs but not iVLCADD2-CMs (Figure 2D–F).

### 2.7. Blocking of lcFAO Flux and Consequently Reducing Accumulation of lcFAO Intermediates Mitigated Electrophysiological Abnormalities in VLCADD-CM

**Figure 3:**
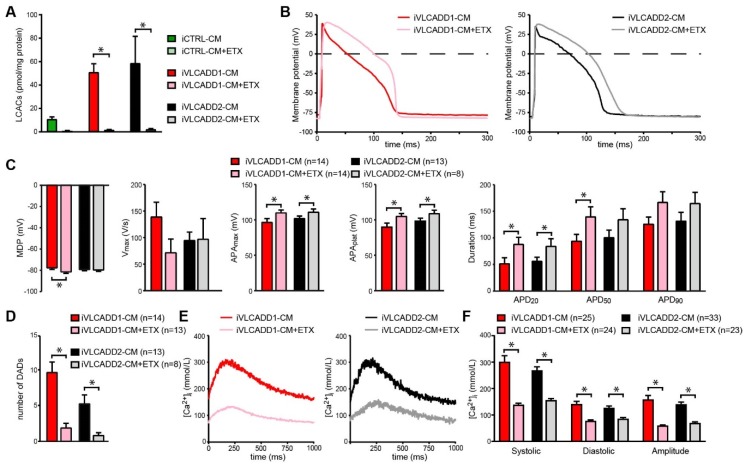
Rescue of electrophysiological abnormalities in iVLCADD1-CMs and iVLCADD2-CMs after pre-incubation with Etomoxir (ETX; 100 µmol/L). (**A**) Intracellular acylcarnitine levels in iVLCADD1-CMs, iVLCADD2-CMs and iCTRL-CMs after ETX incubation for 96 h. Sum of LCAC (C12-, C14-, C14:1-, C16-, C16:1-, C18-, C18:1-carnitine). Values represent mean + SD in two biological replicates of the different hiPSC-CM lines. Statistical analysis was performed using a two-way ANOVA with Tukey’s multiple comparisons test. * = *p* < 0.05. (**B**) Representative APs measured in iVLCADD1-CMs and iVLCADD2-CMs after pre-incubation with ETX or vehicle. (**C**) Average AP parameters after pre-incubation with ETX or vehicle. (**D**) Number of DADs in absence and presence of ETX. (**E**) Representative Ca^2+^ transients of Indo-1 loaded hiPSC-CMs paced at 1 Hz in presence of ETX. (**F**) Average [Ca^2+^]i after ETX or vehicle pre-incubation. For AP analyses and [Ca^2+^]_i_ data is indicated as mean+SEM. * = *p* < 0.05. Statistical analysis was assessed with one-way ANOVA with Tukey’s multiple comparisons test.

The rescued electrophysiological phenotype by resveratrol in iVLCADD1-CMs but not iVLCADD2-CMs strongly suggests that the electrophysiological and [Ca^2+^]_i_ improvement is consequent to improved lcFAO flux. In the next experiments we investigated the effect of lowering the LCAC accumulation without increase of the lcFAO flux on the AP characteristics and Ca^2+^-homeostasis. We used etomoxir to inhibit the production of LCAC by CPT1, which in parallel leads to a further block of the lcFAO flux. Pre-incubation with etomoxir for 48 h led to a complete reduction of intracellular LCAC accumulation in both iVLCADD1-CMs and iVLCADD2-CMs (Figure 3A). This reduction was combined with a concomitant increase in AP amplitudes and APD_20_ as well as a reduced occurrence of DADs (Figure 3B–D). Also the increase in [Ca^2+^]_i_ was reduced in both lines (Figure 3E,F).

## 3. Discussion

Here, we study hiPSC-derived cardiomyocytes of patients with VLCADD and examined two putative pathophysiological mechanisms that have been proposed for the cardiac phenotype: (1) energy shortage due a lower lcFAO flux, and, (2) accumulation of lcFAO intermediates. First, we found that VLCADD-CMs of two unrelated VLCADD patients displayed severe electrophysiological abnormalities compared to control CMs: a shortening in AP duration, a marked increase in DADs and an increased systolic and diastolic Ca^2+^ concentration. Secondly, improvement of all these electrophysiological derangements was possible by incubation with resveratrol, however only in iVLCADD1-CMs, in accordance with the increase of lcFAO flux by resveratrol in fibroblasts of this patient (only in Patient 1 and not in Patient 2), Thirdly, a reduction of lcFAO intermediates accumulation without increasing lcFAO flux, through counterintuitive substrate reduction therapy with etomoxir, was sufficient to restore the majority of the normal AP characteristics and [Ca^2+^]_i_ in VLCADD-CMs. We hence conclude that accumulation of lcFAO intermediates is the mechanism underlying the disturbed AP characteristics and arrhythmia propensity observed in VLCADD.

In VLCADD-CMs derived from two unrelated patients we have documented severe electrophysiological abnormalities (AP shortening, DADs) that likely explain, at least in part, the cardiac arrhythmias observed in VLCADD patients. DADs are caused by spontaneous Ca^2+^ release from the sarcoplasmic reticulum, which typically occurs in Ca^2+^-overload conditions [27]. The higher diastolic [Ca^2+^]i we observed in VLCADD-CMs is consistent with the increased occurrence of DADs, while the higher systolic [Ca^2+^]_i_ may be an explanation for the observed AP shortening via increased calcium-induced L-type Ca^2+^ current (I_Ca,L_) inactivation and increased K^+^ currents [28,29]. In addition, it was previously shown that intracellularly delivered LCACs suppress the I_Ca,L_ density [30], which may further contribute to the found AP shortening. AP shortening facilitates the maintenance of reentrant arrhythmias [31] and DADs may cause triggered activity [19,32,33].

VLCADD patients can have a very heterogeneous clinical outcome [7]. Fibroblasts from Patient 1 in our study exhibited a residual lcFAO flux of 32% of control values, a cellular evaluation that could agree with the development of a mild phenotype [7,34]. In line with this, Patient 1, never presented with a cardiac phenotype or ECG abnormalities throughout life, although she followed since diagnosis a diet with limited periods of fasting. Still, the iVLCADD1-CMs showed severe electrophysiological derangements. A reason for this might be that hiPSC-CMs are known to present with a still immature phenotype under many aspects, therefore these cells may have a less developed mitochondrial system for FAO flux compared to adult cardiomyocytes [35]. Considering this, in the VLCADD-CMs the lcFAO flux may be relatively low due to both immaturity of the cells in combination with the *ACADVL* mutation. Perhaps in the immature hiPSC-CMs, this combination of events causes a more severe phenotype in single hiPSC-CMs than that observed in these individual patients.

In iVLCADD1-CMs both AP abnormalities and the derangements in Ca^2+^ homeostasis were prevented completely by resveratrol. The effects of resveratrol on lcFAO flux are the result of an increased amount of mutated VLCAD protein with residual activity in VLCADD fibroblasts [21]. With this in mind, it was expected that resveratrol would have had no effect effects on LCAC accumulation or electrophysiological parameters in iVLCADD2-CM (homozygous for a deletion mutation with no residual activity).

Incubation with etomoxir resulted in complete reduction of LCAC concentration in both VLCADD-CM lines. Without LCAC production by CPT1, lcFAO flux will be completely blocked, since transport of long-chain acyl-CoAs to the mitochondria is hampered. Still, the AP characteristics as well as [Ca^2+^]_i_ homeostasis were improved. These observations suggest that lcFAO intermediate accumulation (i.e., LCACs or intramitochondrial long-chain acyl-CoAs) underlies the pro-arrhythmia defects in VLCADD-CMs. This is further supported by previous studies in which exogenous addition of large amounts of LCACs to isolated hearts led to anomalies in the electrophysiological properties of cardiomyocytes [12,15].

Our data also provide potential explanations for the hypertrophic cardiomyopathy that is observed in some patients. Indeed, elevated [Ca^2+^]_i_ is involved in the development of hypertrophic cardiomyopathy caused by sarcomere mutations [36,37]. It is hence tempting to speculate that the disturbed [Ca^2+^]_i_ homeostasis we observed in VLCADD-CMs may underlie the cardiomyopathy in patients with VLCADD.

A potential limitation of our study is that resveratrol and etomoxir also have acute effects on cardiac electrophysiology and Ca^2+^ homeostasis [29,38]. However, in our study the cells were pre-incubated and resveratrol and etomoxir were absent during measurement, therefore excluding direct and acute effects on molecules regulating cardiac electrophysiology and Ca^2+^ homeostasis. In agreement with this, we only observed an effect of resveratrol in iVLCADD1-CM and not iVLCADD2-CM, which would have been expected if the effect of resveratrol were independent of VLCADD.

Based on our findings we conclude that accumulation of lcFAO intermediates is important in development of arrhythmias in VLCADD, hence a strategy to prevent lcFAO intermediate accumulation in patients is required. One of the current treatment strategies of lcFAODs is replacing dietary long-chain triglycerides (LCT) with medium-chain length triglycerides (MCT). Theoretically this would lead to less lcFAO intermediate production, since the enzymes responsible for medium chain fatty acid oxidation are functioning normally in individuals with lcFAODs. However, it should be noted that contrasting data have been reported on the effects of MCT on LCAC accumulation. On the one hand, a significant post-exercise lowering of LCACs in plasma was found after MCT in children with lcFAOD [39]. On the other hand, other studies show that in lcFAOD mice [40], human lcFAOD fibroblasts [41] and in premature infants without a lcFAOD [42], MCT can be elongated to LCT and even lead to higher LCAC accumulation [43]. Taken together these studies suggest that dietary modification with MCT in lcFAODs disorders has to be carefully adapted to energy demand in order to prevent lcFAO intermediate accumulation.

In future studies it would be interesting to test the effect of medium-chain fatty acid supplementation to the culture medium of VLCADD-CMs as well as supplementation of other current treatment modalities such as carnitine and triheptanoin [2] on the electrophysiological derangements in VLCADD-CM.

In conclusion, we provide insight into cardiomyocyte electrophysiological abnormalities underlying VLCADD and provide compelling evidence that pharmacologically reduced accumulation of lcFAO intermediates, either by enhancement (resveratrol) or blockage (etomoxir) of lcFAO flux, rescues the cellular arrhythmic phenotype in VLCADD cardiomyocytes, also in cells of a patient with a very severe VLCADD. This finding potentially impacts on the treatment of patients with VLCADD.

## 4. Materials and Methods

### 4.1. Patient Selection and Clinical Data Analysis

Two patients with genetically confirmed VLCADD were recruited at the Dutch FAO Expertise Center in Utrecht. Primary fibroblasts were already available for diagnostics. Written informed consent for the use of patient data and fibroblasts was obtained from both patients.

### 4.2. Fibroblast Culture

Fibroblasts derived from VLCADD patients and healthy controls were maintained in a humidified atmosphere containing 5% CO_2_ in HAM F-10 medium (Gibco) supplemented with 10% fetal bovine serum (Gibco) and antibiotics (100 µg/mL streptomycin, 100 U/mL penicillin and 250 µg/mL amphotericin) [44].

### 4.3. VLCAD Activity

VLCAD activity in lymphocytes and fibroblasts was measured using palmitoyl-CoA as substrate as described previously [25]. Data is expressed as nmol per minute per mg protein and percentage of reference mean that was valid at time of analysis.

### 4.4. Measurement of Long-Chain Fatty Acid Oxidation (lcFAO) Flux

Skin fibroblast were plated in 48-well plates. Long-chain fatty acid oxidation was measured by the production of ^3^H_2_O from [9–^3^H(N)]-oleic acid as described previously [45]. Measurements were done at 37 °C in duplicate and lcFAO flux is expressed as percentage of mean activity of skin fibroblasts of healthy controls measured in the same experiment.

### 4.5. Acylcarnitine Profiling

Fibroblasts were cultured in 12-well plates and incubated for 96 h at 37 °C in MEM medium with 0.4 mmol/L L-carnitine, 4 g/L bovine serum albumin (BSA) and 120 µmol/L of [U−^13^C]-palmitate. After 96 h incubation medium was collected. Cells were washed with PBS and harvested with 0.4 mmol/L NaOH. Protein levels were determined using bicinchoninic acid (BCA) assay with human serum albumin (HSA) in 0.4 mmol/L NaOH used as a standard. Collected medium was deproteinized using acetonitrile and subsequently butyl esters were formed. The dried butyl esters were dissolved in acetonitrile and analyzed using tandem mass spectrometry. ^2^H_3_-C3-carnitine, ^2^H_3_-C8 carnitine, ^2^H_3_-C16 carnitine were used as internal standards. For intracellular acylcarnitine profiling in the hiPSC-CMs, cells were cultured on RPMI 1640 plus insulin-containing B27 supplement for 96 h. Cells were harvested with trypsin and cell pellets were extracted in acetonitrile with internal standards and measured on high performance liquid chromatography mass spectrometry by a Q-ExactiveTM mass spectrometer (Thermo Scientific).

### 4.6. Generation and Maintenance of hiPSC Lines

Dermal fibroblasts of two VLCADD patients were expanded and reprogrammed to hiPSCs by Sendai virus (CytoTune-iPS 2.0 Sendai Reprogramming Kit, #A16517,Thermo Fisher, Waltham, MA, USA, according to manufacturer’s instructions) -mediated delivery of genes, specifically *OCT4*, *KLF4*, *c-MYC*, *SOX2*, as previously described [46]. Fibroblasts from both patients and from a healthy control (a 22 year old healthy male) were reprogrammed to generate hiPSC lines (iVLCADD1 from Patient 1, iVLCADD2 from Patient 2 [46] and iCTRL [47,48] (Figure S1)). The experiments were performed on one hiPSC line per individual (named iVLCADD1 and iVLCADD2) after verification of their karyotype integrity (Figure S1A), pluripotency genes expression (Figure S1B,C) and differentiation potential (Figure S1D,E). A previously characterized skin fibroblasts-derived hiPSC line was used as control (iCTRL) [48]. All lines were maintained in mTeSR1 chemically defined medium on matrigel coated cell culture vessels and passaged using 0.5 mmol/L EDTA.

### 4.7. Differentiation of hiPSC Lines into Cardiomyocytes

All lines were differentiated in the presence of RPMI medium (Gibco) supplemented with B27 (GIBCO), in absence of insulin from day 1 to day 7 (with the addition of the molecules described below) and in presence of insulin from day 8 to day 30. Differentiation started when the hiPSC cultures were 70–90% confluent. Mesoderm was induced using CHIR99021, Activin A and BMP4, followed by Wnt-pathway inhibition by 5 µmol/L IWP4 (modified from Dambrot et al. [49]). Since these hiPSC lines appeared quite sensitive to the growth factors treatment as published (i.e., high mortality) we applied a specific modulation of the concentration and duration of treatment with these factors to generate the optimal conditions for their highest survival and differentiation into cardiomyocytes. Concentrations of these factors and duration of their stimulation were adapted in a hiPSC line-specific manner as indicated. For the control hiPSC line (iCTRL): 1.5 µmol/L CHIR99021, 20 ng/mL Activin A, 20 ng/mL BMP4 on day 1, no factors on day 2, IWP4 on day 3-5; for the iVLCADD1 line: 1.5 µmol/L CHIR99021, 10 ng/mL Activin A, 10 ng/mL BMP4 on day 1, no factors on day 2, 2.5 µmol/L IWP4 on day 3-4; for the iVLCADD2 line: 1.5 µmol/L CHIR99021,10 ng/mL Activin A, 15 ng/mL BMP4 on day1-3, no factors on day 4, 2.5 µmol/L IWP4 on day 5–7. The differentiating hiPSC cultures of all lines were enriched for cardiomyocytes through a previously described metabolic selection [50], by culturing the cells from day 31 to day 36 in glucose-depleted RPMI medium supplemented with 4 mmol/L sodium lactate.

### 4.8. Preparation of hiPSC-CM for Electrophysiology

Lactate-treated hiPSC-CM cultures were detached from the culture vessels (usually 12 wells of a 24-well tissue culture plate) using 5× TrypLE Select Enzyme (Gibco) in PBS for a few minutes at 37 °C. The cells were collected in a 15 mL tube and washed once with PBS to dilute away the enzyme, spun-down at 150× *g* for 5 min. To obtain a dissociation to single cells, the pellet was carefully resuspended in 1 mL Low-Ca^2+^ Tyrode solution (composition as described by Meijer van Putten et al. [51]) and incubated at room temperature for 10 min. Subsequently, Liberase (Roche Chemicals) and Elastase (Serva) were added at a final concentration of 20 µg/mL and 0.94 µg/mL, respectively, as well as 500 µL RPMI medium containing 2% B27 supplement. The cells were then incubated overnight (for 10–12 h) at 37 °C in presence of 5% CO_2_. After the incubation the cells suspension was diluted to 6 mL with RPMI medium containing 2% B27 supplement and centrifugated at 160× *g* for 5 min. The pellet was gently resuspended in 600 µL RPMI medium containing 2% B27 supplement and 50 U/mL penicillin and 50 µg/mL streptomycin and seeded on matrigel-coated glass coverslips lying inside wells of a 24 well plate. Presence of small groups of cells in addition to single cells were tolerated to avoid an excessively harsh dissociation. The seeded cells were let recover at 37°C in presence of 5% CO_2_ for 8–10 days, with medium change every other day, then subject to electrophysiological analysis. Stock solution of resveratrol (Sigma, St. Louis, USA) and etomoxir (Sigma, St. Louis, USA) were prepared in dimethyl sulfoxide (DMSO) and diluted in culture medium to a final concentration of 50 µmol/L resveratrol or 100 µmol/L etomoxir 48 h prior to electrophysiological measurement.

### 4.9. Cellular Electrophysiology in hiPSC-CMs

Data acquisition. Action potentials (APs) were measured using the amphotericin-perforated patch-clamp technique and an Axopatch 200B amplifier (Molecular Devices, Sunnyvale, CA, USA). Voltage control, data acquisition and analysis were realized with custom software. Pipettes (resistance 2–3 MΩ) were pulled from borosilicate glass capillaries (Harvard Apparatus, UK) using a custom-made microelectrode puller. Cell membrane capacitance (Cm) was calculated [51] and potentials were corrected for the calculated liquid junction potential [52]. Signals were low-pass-filtered with a cut-off of 5 kHz and digitized at 40 kHz.

AP measurements. AP were recorded at 36 ± 0.2 °C using an extracellular, Tyrode solution containing (in mmol/l): 140 NaCl, 5.4 KCl, 1.8 CaCl_2_, 1 MgCl_2_, 5.5 glucose, 5 HEPES, pH 7.4 (NaOH). Pipettes were filled with solution containing (in mmol/L): 125 K-gluc, 20 KCl, 5 NaCl, 0.44 amphotericin-B, 10 HEPES, pH 7.2 (KOH). Typically, hiPSC-CMs have a small or even complete lack of the inward rectifying potassium current (IK1) [51]. Consequently, hiPSC-CMs have a depolarized maximal diastolic potential (MDP) and are frequently spontaneously active [53]. We overcome this limitation by injection of an in silico I_K1_ with kinetics of Kir2.1 channels through dynamic clamp, as previously described in detail [51]. Here, we used an amount of 2 pA/pF I_K1_ peak outward current which resulted in quiescent hiPSC-CMs with close-to-physiological resting membrane potential. APs were elicited at 1-Hz by 3-ms, ~1.2× threshold current pulses through the patch pipette. Susceptibility to delayed afterdepolarization (DADs) was tested by applying a 3-Hz pacing episode (10-s) followed by an 8-s pause. After the pause, a single AP was evoked to test the inducibility of early afterdepolarizations (EADs). DADs were defined as depolarizations larger than 1 mV during the 8-s pause. We analyzed MDP, AP amplitude (APA_max_), AP plateau amplitude (APA_plateau_; measured at 20 ms after initiation of the action potential upstroke), maximum AP upstroke velocity (Vmax) and AP duration at 20%, 50% and 90% repolarization (APD_20_, APD_50_ and APD_90_, respectively). Parameters from 10 consecutive APs were averaged. Number of DADs of >1 mV was counted and averaged over 5 recording traces.

### 4.10. Cytoplasmic Calcium Measurements

Cells were prepared for the calcium measurements in a similar manner as described above for the electrophysiology measurements. Intracellular calcium transients were measured in the hiPSC-CMs at 37 °C stimulated at 1 Hz, using a fluorescent probe Indo-1 as described previously [54].

### 4.11. Statistical Analysis

Data are presented as mean±standard error of the mean (SEM) for the electrophysiological data or as mean ± standard deviation (SD) for the biochemical experiments. Statistical analysis was carried out with SigmaStat 3.5 software and Graphpad Prism 7. Normality and equal variance assumptions were tested with the Kolmogorov-Smirnov and the Levene median test, respectively. Two groups were compared with unpaired t-test or, in case of a failed normality and/or equal variance test, Mann-Whitney Rank Sum Test. More than 2 groups were compared using One-Way ANOVA followed by a Student-Newman-Keuls Method post hoc test. *p* < 0.05 was defined as statistical significance. The specific statistical test used are indicated in each figure legend.

## Figures and Tables

**Table 1:** Clinical and biochemical characteristics of patients.

PID (Name of hiPSC Line)	Patient 1 (iVLCADD1)	Patient 2 (iVLCADD2)
Age of presentation (current age)	1.25 y (23 y)	0.1 y (22 y)
Sex	female	female
Mutations in *ACADVL* gene	c. 848T >C (p.Val283Ala) c.1141_1143delGAG (p.Glu381del)	c.104delC (p.Pro35Leufs*26) c.104delC (p.Pro35Leufs*26)
VLCAD activity (in nmol/min/mg and % of controls)	*Fibroblasts:* 0.10 (3%) *Lymphocytes:* 0.18 (5%)	*Fibroblasts:* <0.06 (0%) *Lymphocytes* <0.15 (0%)
lcFAO flux (% of control)	32	7
Maximal Creatine Kinase	400 U/L	99889 U/L
Signs at presentation	Hypoglycemia 0.2 mmol/L Hypothermia (35.9 °C)	Hypoglycemia 1.7 mmol/L, vomiting, convulsions, cardiomyopathy (reversible)
Cardiac history	Age 15y: Holter ECG: Normal conduction.	Age 14y: ECG: aspecific repolarization abnormalities. (flat ST-T segments in the inferior and left lateral leads). Some early repolarizations in the inferior leads. Age 15y: dilated cardiomyopathy (reversible) Age 18y: Holter ECG: single ectopy from the atrial side as well as from the ventricular side. Mobitz II AV-block during sleep.
Other signs or symptoms	None	Rhabdomyolysis Exercise intolerance

## Supplementary Materials

Supplementary materials can be found at https://www.mdpi.com/1422-0067/21/7/2589/s1.
