# Supplementary material for: Electrophysiological Abnormalities in VLCAD Deficient hiPSC-Cardiomyocytes Can Be Improved by Lowering Accumulation of Fatty Acid Oxidation Intermediates
Source: Int J Mol Sci. 2020 Apr 8;21(7):2589. doi: 10.3390/ijms21072589 (PMC7177397; doi:10.3390/ijms21072589)
Supplement: Supplementary file 1 [file ijms-21-02589-s001.pdf]

Supplementary Table 1 Primer sequences pluripotency markers

Primer	Primer forward	Primer reverse	Length (bps)
hGAPDH	AGAGGCAGGGATGATGTTCT	TCTGCTGATGCCCCCATGTT	258
hSOX2	ATG CAC CGC TAC GAC GTG A	CTT TTG CAC CCC TCC CAT TT	437
hGDF3	TTCGCTTTCTCCCAGACCAAGGTTTC	TACATCCAGCAGGTTGAAGTGAACAGCACC	311
hOCT4	GACAACAATGAAAATCTTCAGGAGA	TTCTGGCGCCGGTTACAGAACCA	218
hFOXD3	GTGAAGCCGCCTTACTCGTAC	CCGAAGCTCTGCATCATGAG	353
hLIN28	AGTAAGCTGCACATGGAAGG	ATTGTGGCTCAATTCTGTGC	410
hNANOG	AGTCCCAAAGGCAAACAACCCACTTC	ATCTGCTGGAGGCTGAGGTATTTCTGTCTC	164

Supplementary Table 2 Primer sequences for rt-PCT germ layer markers

Primer	Primer forward	Primer reverse	Length (bps)
hNANOG	AGTCCCAAAGGCAAACAACCCACTTC	ATCTGCTGGAGGCTGAGGTATTTCTGTCTC	164
hAFP	ACTCCAGTAAACCCTGGTGTTG	GAAATCTGCAATGACAGCCTCA	255
hALB	CCTTTGGCACAATGAAGTGGGTAACC	CAGCAGTCAGCCATTTCACCATAGG	355
hα-MHC	GTCATTGCTGAAACCGAGAATG	GCAAAGTACTGGATGACACGCT	413
hCTNT	GACAGAGCGGAAAAGTGGGA	TGAAGGAGGCCAGGCTCTAT	305
hTH	GCGGTTCATTGGGCGCAGG	CAAACACCTTCACAGCTCG	215
hGAPDH	AGAGGCAGGGATGATGTTCT	TCTGCTGATGCCCCCATGTT	258

Supplementary Table 3 List of antibodies

Primary antibodies

Antigen	Host	Dilution	Blocking	Provider
AFP	Rabbit (IgG)	IF: 1:100	IF: 1% BSA/DPBS	Dako #A0008-4oC
LIN28	Goat (IgG)	IF: 1:300	IF: 1% BSA/DPBS	R&D systems #AF3757
NANOG	Goat (IgG)	IF: 1:200	IF: 1% BSA/DPBS	Abcam #PA5-18406
OCT4	Goat (IgG)	IF. 1:40	IF: 1% BSA/DPBS	R&D systems #AF1759
SOX2	Mouse (IgG)	IF: 1:50	IF: 1% BSA/DPBS	R&D systems #MAB2018
SSEA-4	Mouse (IgG)	IF: 1:200	IF: 1% BSA/DPBS	Abcam #MC813
α-SMA	Mouse (IgG2A)	IF: 1:3000	IF: 1% BSA/DPBS	Sigma-Aldrich #A2547
β-III-TUBULIN	Mouse (IgG2A/K)	IF: 1:2000	IF: 1% BSA/DPBS	Covance #MMS-435P

α-SMA: α- smooth muscle actin, SSEA4: stage-specific embryonic antigen 4

Supplementary Table 4

Secondary antibodies

Fluorophore & Antigen	Host	Dilution	Company
Alexa Fluor 488-anti-mouse	Donkey (IgG)	1:1000	Thermo Fisher Scientific #A21202
Alexa Fluor 555-anti-goat	Donkey (IgG)	1:1000	Thermo Fisher Scientific #A21432
Cy3-anti-mouse	Goat (IgG+IgM)	1:300	Jackson ImmunoResearch #115-165-068
Cy3-anti-rabbit	Goat (IgG)	1:600	Jackson ImmunoResearch #111-165-003
FITC-anti-mouse	Goat (IgM)	1:100	Jackson ImmunoResearch #115-096-072

Figure S1

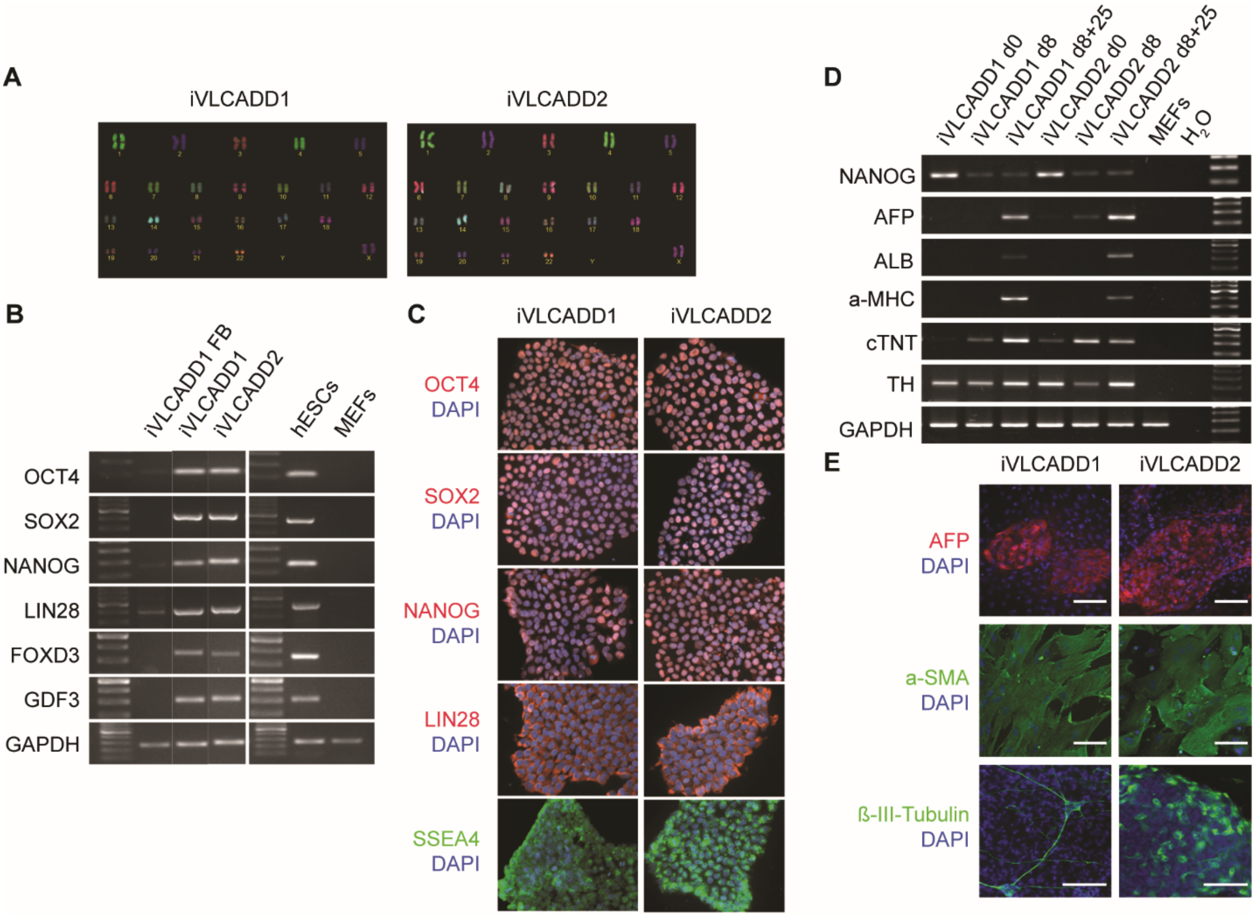

Figure S1: Pluripotency characterization of patient-specific iPSC lines generated from dermal fibroblasts. A. Karyotype of lines iVLCADD1and iVLCADD2 analyzed by COBRA [53] B. Expression of endogenous pluripotency markers (*OCT4, SOX2, NANOG, LIN28, FOXD3, GDF3*) in human iPSC lines (iVLCADD1; iVLCADD2) compared to primary fibroblasts (VLCADD1 FBs) assessed by reverse transcriptase-PCR; human embryonic stem cells (hESCs) were used as positive control; mouse embryonic fibroblasts (MEFs) were used as negative control. C. Immunofluorescence staining for pluripotency markers (OCT4, SOX2, NANOG, LIN28, SSEA4); DNA is stained with DAPI (blue); scale bar, 100 µm. D. Transcript expression of characteristic genes of different germ layers: AFP (alpha-fetoprotein) and ALB (albumin) for endoderm, α-MHC (alpha-myosin heavy chain) and cTNT (cardiac troponin T) for mesoderm, TH (tyrosin hydroxylase) for ectoderm during spontaneous differentiation of the two iVLCADD1 and iVLCADD2 lines at day 8 and day 33 of differentiation. MEFs are used as negative control.E. Immunofluorescence staining for AFP (endoderm), α-SMA (alpha smooth muscle actin, for mesoderm), β-III-tubulin (neuroectoderm) in both hiPSC lines. Scale bar, 100 µm.

Figure S2

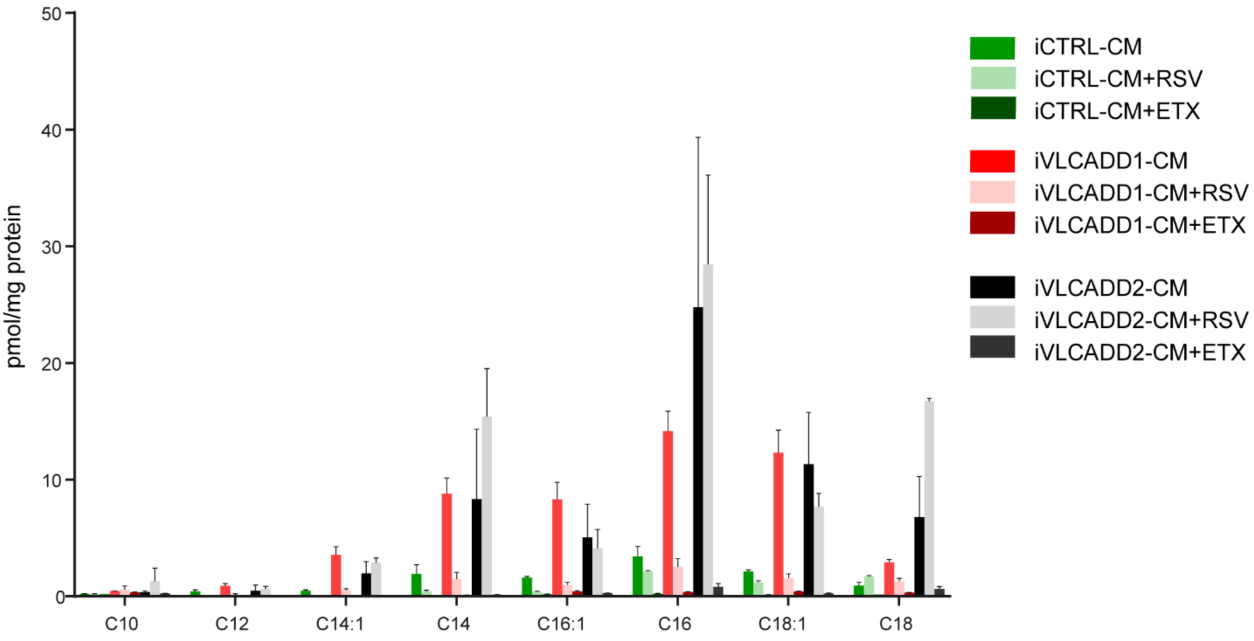

Figure S2: Different long-chain acyl-carnitine species measured in the hiPSC-CMs. Intracellular acylcarnitine levels in iVLCADD1-CMs, iVLCADD2-CMs, and iCTRL-CMs after etomoxir (ETX), resveratrol (RSV) or vehicle (DMSO) incubation for 96 hours. Acylcarnitines were measured in cell pellets and normalized for total amount of protein. The x-axis shows the different acylcarnitine carbon chain lengths that were measured. Values are presented as the mean of two biological replicates with SD.

Figure S3

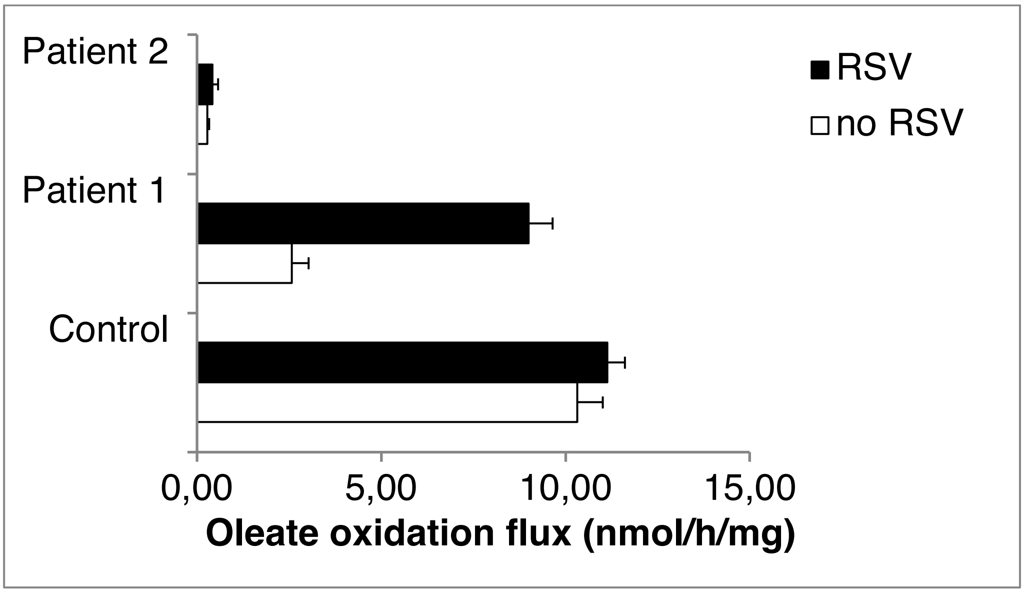

Figure S3. Long-chain fatty acid oxidation flux measured in cultured fibroblast of Patient 1 and Patient 2 and two healthy controls incubated with or without resveratrol (RSV) in the medium. RSV increases lcFAO flux in Patient 1 (carrying missense mutation and 3-bp deletion) but not in Patient 2 (homozygous for deletion mutation)
